# Safety of Physical Activity After Cervical Artery Dissection

**DOI:** 10.1111/ene.70548

**Published:** 2026-02-25

**Authors:** Lukas Mayer‐Suess, Josefin Kaufmann, Christopher Traenka, Silvia Felicetti, Sebastian Kaufmann, Benjamin Dejakum, Lukas S. Enz, Gudrun Ratzinger, Simon Truessel, Elke R. Gizewski, Stefan Kiechl, Stefan Engelter, Michael Knoflach

**Affiliations:** ^1^ Department of Neurology Medical University of Innsbruck Innsbruck Tyrol Austria; ^2^ Department of Neurology and Stroke Centre University Hospital Basel and University of Basel Basel Switzerland; ^3^ Department of Rehabilitation and Neurology University Department of Geriatric Medicine FELIX PLATTER Basel Switzerland; ^4^ Department of Clinical Research University of Basel Basel Switzerland; ^5^ Department of Dermatology, Venereology and Allergology Medical University of Innsbruck Innsbruck Tyrol Austria; ^6^ Department of Radiology Medical University of Innsbruck Innsbruck Tyrol Austria; ^7^ Neuroimaging Research Core Facility Medical University of Innsbruck Innsbruck Tyrol Austria; ^8^ VASCage, Research Center on Vascular Ageing and Stroke Innsbruck Tyrol Austria

**Keywords:** cervical artery dissection, outcome, physical activity, recurrence, sports, stroke

## Abstract

**Background:**

Cervical artery dissection (CeAD) is a major cause for stroke in young adults. A timely association with minor‐to‐moderate unimposing cervical trauma, which is often sports‐related, is common in CeAD. Our goal was to assess whether physical activity puts patients at risk post‐CeAD.

**Methods:**

Pooled data from two prospective observational CeAD cohorts with in‐person follow‐up of at least 1‐year post‐CeAD were assessed. Changes in physical activity were recorded using patient‐reported assessment of change in activity compared to pre‐CeAD. Baecke score‐derived sports index was applied to address the association between physical activity intensity and our outcomes. Outcomes were (1) recurrent dissection and (2) cerebral ischemia upon follow‐up.

**Results:**

A total of 648 CeAD patients were recorded. Physical activity‐specific follow‐up data were available in 333 (59.7%). The median follow‐up duration was 6.5 (IQR 3.1, 10.9) years with 17/333 (5.1%) suffering CeAD recurrence and 22/33#3 (6.6%) experiencing cerebral ischemia. A total of 197 of 333 (59.2%) patients reported a change in physical activity post‐CeAD (127 [64.5%] decrease, 70 [35.5%] increase). Neither overall change, increase, or decrease of physical activity was associated with recurrent CeAD or cerebral ischemia (*p* > 0.2 throughout). However, regular performance of higher‐intensity sports, assessed via Baecke score‐derived sports index, associated in trend to dissection recurrence (OR 3.43 [0.86, 13.64]; *p* = 0.080).

**Conclusions.:**

CeAD patients should be reassured that regaining physical activity after CeAD is safe. However, moderation on exertion should be discussed on an individual patient basis.

## Graphical abstract

We assess whether cervical artery dissection (CeAD) patients change their physical activity post‐CeAD and whether such changes or the intensity of physical activity performed relate to dissection or stroke recurrence. In this pooled registry analysis of 333 patients with 6.5 years of follow‐up, 59.2% changed activity levels, with 64.5% and 35.5% reporting decrease or increase, respectively. Neither overall change, increase, nor decrease was associated with CeAD or stroke recurrence; however, regular performance of higher‐intensity sports showed a tendency toward higher risk of CeAD recurrence.

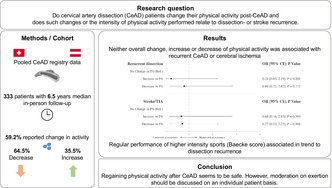

## 1 Introduction

Cervical artery dissection (CeAD) is one of the main causes for stroke in young adults and typically occurs spontaneously in otherwise healthy individuals [1, 2, 3]. Up to 40% of CeAD patients report minor‐to‐moderate unimposing cervical trauma attained during day‐to‐day activities prior to dissection diagnosis, with a relevant part of those being sports‐related [4, 5]. Case reports have suggested an association between different types of sports, such as golf, scuba diving, or combat sports, and the occurrence of CeAD [6, 7, 8]. It is well recognized that physical activity positively affects well‐being and overall stroke risk. In CeAD patients, where sports are hypothesized to potentially be causally linked, the fear of recurrence may hamper a patient's recuperation of a previously active lifestyle [9, 10]. As it is hitherto unknown if the associations between physical activity and CeAD occurrence do exist, medical personnel are unable to give evidence‐based recommendations concerning the safety of resuming sporting activities post‐CeAD to these young patients [8, 11, 12].

### 1.1 Aims and Hypothesis

It was our goal to evaluate if CeAD patients tend to change their physical activity after CeAD and whether such changes or intensity of physical activity relate to CeAD recurrence. In assessing the association between physical activity and CeAD recurrence, we aim to assist clinicians in advising CeAD patients in the future.

## 2 Methods

### 2.1 Patient Recruitment and Selection

This study is a collaborative two‐center effort of two stroke centers specialized in CeAD care. It encapsulates the prospective ProSect registry of the Medical University of Innsbruck and the data from the CeAD registry of the Stroke Center at the University Hospital Basel, Switzerland. ProSect and Basel's registry collect data on consecutive CeAD patients treated at their respective centers from 1995 and 1999 onward, respectively. The registries prospectively followed CeAD patients through in‐person long‐term follow‐up. Details of the involved registries have been published previously [9, 13, 14, 15, 16, 17, 18, 19]. In all ProSect patients, CeAD was verified through visualization of mural hematoma in fat‐saturated T1‐MRI images. The Basel CeAD registry established the diagnosis of CeAD based upon established and widely accepted diagnostic CeAD criteria including the following arterial findings (at least one): presence of a mural hematoma, aneurysmal dilation, long tapering stenosis, intimal flap, double lumen, or occlusion situated > 2 cm above the carotid bifurcation, revealing an aneurysmal dilation or a long tapering stenosis after recanalization [1, 2, 19].

To be eligible for the current analysis, CeAD diagnosis had to be verified through MRI, and in‐person follow‐up of at least 1‐year post‐CeAD had to be available. The applied registries harmonized and pooled data of all recruited patients encompassing patient characteristics (age; biological sex; pre‐existing conditions; recent respiratory infection or minor trauma prior to CeAD; antithrombotic treatment at discharge and follow‐up), clinical presentation (ischemic stroke and/or TIA; local symptoms), functional as well as clinical scales (modified Rankin scale [mRS] pre‐CeAD as well as at admission and follow‐up; National institutes of Stroke Scale [NIHSS] at admission if stroke occurred), CeAD‐related imaging characteristics (CeAD‐affected vessel; single or multiple vessels affected at baseline; CeAD‐related vessel pathology) and follow‐up characteristics (recurrent CeAD and/or stroke).

In terms of study specific values, we recorded patient‐reported changes in physical activity. To be eligible, patients had to have at least a 1‐year post‐CeAD in‐person follow‐up. In a subset of ProSect registry patients (Innsbruck only), the Baecke questionnaire at follow‐up additionally captured intensity of physical activity and information on reason for change in physical activity was available [20].

### 2.2 Variable Definition

Date of hospital admission due to CeAD was defined as baseline, while date of last in‐person assessment in which physical activity was assessed in‐person was considered follow‐up. Clinical presentation at baseline included evidence of cerebral ischemia (ischemic stroke, retinal infarction, or transient ischemic attack [TIA]) as well as local symptoms (head/neck pain, Horner's syndrome, tinnitus, and cranial nerve palsy) and was adjudicated by a stroke physician. TIA was defined as transient deficits due to ischemia without evidence of cerebral infarction (tissue‐based definition). Minor trauma was defined as recent head/neck trauma without concomitant internal or external signs of injury, which patients also did not seek medical care for [4]. Recent infection was considered if respiratory infection ≤ 4 weeks prior to CeAD‐related admission was reported [4]. Clinical and functional status of patients were evaluated using the NIHSS and the mRS. Further, prior debilitating deficit was defined as a medical diagnosis that has an impact on mobility prior to CeAD occurrence, as adjudicated by experienced neurologists. Imaging characteristics included CeAD localization (vertebral or internal carotid artery), evidence of multiple vessel CeAD (defined as more than one CeAD in imaging upon admission), as well as vessel pathology type (occlusion, stenosis). Type of antithrombotic treatment was recorded at CeAD‐related hospital discharge as well as at follow‐up and defined as either intake of oral anticoagulation (vitamin K antagonists or direct oral anticoagulants), antiplatelets or none.

Changes in physical activity were assessed through structured in‐person interviews performed by study personnel. All included patients were asked to compare their overall mean estimated physical activity level after CeAD to prior to suffering CeAD. Additionally, patients were asked to record three physical activities that they regularly performed during the follow‐up time. All reported activities were graded in a three‐level system concerning the physical exertion and in accordance with literature [20] (1): low intensity—energy expenditure of 0.76 MJ/h (2); medium intensity—average energy expenditure of 1.26 MJ/h and (3) high intensity—average energy expenditure of 1.76 MJ/h. In addition to the activity type, the duration per week (in hours) as well as the months per year that said activity is performed was recorded to derive a modified Baecke score dependent sports index. The Baecke score sports index is considered a measure of physical activity intensity of regularly performed sporting activities with more detailed information previously published [20]. A description of modified sports index value derivation within our study is presented in Figure S1. In short, duration (hours per week) and regularity (months per year) are recorded and multiplied with measures of physical exertion for each individual activity reported. In our case, patients reported three individual sporting activities, resulting in three individual sport values which were summed and divided by 3 to establish a mean Baecke score‐derived sports index. Our predefined outcomes were [1] recurrent dissection, considered if new mural hematoma through evidence of T1‐fat‐saturated hyperintensity in cervical arteries was visualized ≥ 1 month after initial CeAD and [2] cerebral ischemia upon follow‐up representing a composite of symptomatic ischemic stroke and silent ischemic infarction visualized in routine imaging or TIA.

### 2.3 Statistical Methodology

Differences in categorical or continuous variables were calculated using the Pearson Chi‐Square or Mann–Whitney U‐test, respectively. Logistic regression analysis, applying adjustment for confounders (ischemia at baseline, sex, mRS at admission, CeAD related vessel pathology, length of follow‐up, and age), was used to assess the association of change in physical activity to our outcomes. A dose–response relationship assessment between sport of highest exertion level performed and occurrence of CeAD recurrence was performed. Herein, an individual was grouped to either level of exertion 1, 2 or 3 in accordance with the single highest exertion level rated sport performed (as described above), independent of duration (i.e., sole performance of a high‐intensity sports type). Further, a dose–response relationship between Baecke score‐derived sports index and recurrent CeAD was calculated, enabling the inclusion of duration spent in various levels of exertion. Upon visualization, sports index values were categorized from 1 to 4, with decimal points being rounded to the nearest whole number. P values were two‐sided, and an alpha level of 0.05 is used. Analysis was conducted using IBM SPSS Statistics (IBM Corp. Released 2023. IBM SPSS Statistics for Windows, Version 29.0.2.0 Armonk, NY: IBM Corp).

### 2.4 Standard Protocol Approvals, Registration, and Patient Consents

All analyses were approved by the local ethics committee at the Medical University Innsbruck (EK#UN5072,325/4.1) and of Basel (EKNZ PB‐2016‐00547). Appropriate informed consent of included patients was obtained.

## 3 Results

A total of 648 CeAD patients were recorded, with 558 (86.1%) being eligible for the present analysis (i.e., an initial CeAD diagnosis made by MRI and a minimum of 1‐year follow‐up). Follow‐up data on physical activity were available for 333 (59.7%) patients, which were considered our study cohort. Differences between those with and without physical activity data available are given in Table S‐1. In short, patients with data on physical activity unavailable had a lower frequency of migraine (16.9% vs. 28.2%) and local symptoms (80.9% vs. 90.4%) as well as a higher rate of vertebral artery dissections (49.8% vs. 35.4%) and longer follow‐up duration (8.3 [3.7, 12.8] vs. 6.5 [IQR 3.1, 10.9] years median follow‐up). Of 333 participants, 197 (59.2%) indicated that their level of activity changed upon long‐term follow‐up, with 127 of 197 (64.5%) reporting a decrease and 70 of 197 (35.5%) an increase in activity (Table 1).

**TABLE 1:** Differences between those with overall change in physical activity as well as dependent on increase or decrease in physical activity.

	Physical activity data available			Change in physical activity		
	*No change in PA*	*Change in PA*	*P‐Value*	*Decrease in PA*	*Increase in PA*	*P‐Value*
*N*	136 (40.8)	197 (59.2)		127 (64.5)	70 (35.5)	
***Patient characteristics***						
Age, years^b^	46.2 (40.0, 52.1)	45.4 (37.2, 52.7)	0.423	46.6 (38.0, 53.9)	43.8 (34.2, 49.7)	0.022
Male sex^a^	76 (55.9)	115 (58.4)	0.367	76 (59.8)	39 (55.7)	0.340
mRS prior to CeAD^b^	0.0 (0.0, 0.0)	0 (0.0, 0.0)	0.821	0 (0.0, 0.0)	0 (0.0, 0.0)	0.925
Migraine^a^	40 (29.4)	54 (27.4)	0.370	34 (26.8)	20 (28.6)	0.471
Hypertension^a^	36 (26.5)	67 (34.0)	0.086	44 (34.6)	23 (32.9)	0.476
Dyslipidemia^a^	33 (24.3)	40 (20.3)	0.230	28 (22.0)	12 (17.1)	0.281
***Clinical presentation***						
Cerebral ischemia^a^	77 (56.6)	134 (68.0)	0.016	87 (68.5)	47 (67.1)	0.532
TIA only^a^	21 (15.4)	23 (11.7)	0.212	18 (14.2)	5 (7.1)	0.118
NIHSS admission^b^	2.0 (0.0, 3.0)	2.0 (0.0, 7.0)	0.021	2.5 (1.0, 8.0)	2.0 (0.0, 7.0)	0.534
mRS admission^b^	1.0 (1.0, 2.0)	1.0 (0.0, 2.0)	0.040	2.0 (0.0, 2.0)	1.0 (1.0, 2.0)	0.503
Local symptoms^a^	128 (94.1)	173 (87.8)	0.181	110 (86.6)	63 (90.0)	0.453
Minor trauma^a^	36 (26.5)	54 (27.4)	0.458	38 (29.9)	16 (22.9)	0.283
Recent respiratory infection^a^	28 (20.6)	34 (17.3)	0.273	19 (15.0)	15 (21.4)	0.159
Multiple vessel CeAD^a^	22 (16.2)	28 (14.2)	0.352	18 (14.2)	10 (14.3)	0.555
Vertebral artery CeAD^a^	51 (37.5)	66 (33.5)	0.665	38 (29.9)	28 (40.0)	0.119
Pathology type^a^			0.014			0.843
Occlusion	36 (26.5)	72 (36.5)		47 (37.0)	25 (35.7)	
Stenosis	75 (55.1)	88 (44.7)		57 (44.9)	31 (44.3)	
***Treatment***						
Medication at discharge^a^			0.239			0.581
Antiplatelets	76 (55.9)	94 (47.7)		63 (49.6)	31 (44.3)	
Oral anticoagulants	58 (42.6)	100 (50.8)		62 (48.8)	38 (54.3)	
Medication follow‐up^a^			0.072			0.882
Antiplatelets	67 (49.3)	122 (61.9)		80 (63.0)	42 (60.0)	
Oral anticoagulants	5 (3.7)	7 (3.6)		5 (3.9)	2 (2.9)	
Days of hospital stay^b^	7 (3.0, 12.0)	9 (5.0, 12.0)	0.010	9 (5.0, 13.0)	9 (5.0, 12.0)	0.878
***Follow‐up***						
Duration, years^b^	6.1 (2.9, 11.1)	6.6 (3.2, 10.8)	0.952	6.3 (2.9, 10.4)	7.6 (3.7, 11.3)	0.261
Recurrent CeAD^a^	8 (5.9)	9 (4.6)	0.384	7 (5.5)	2 (2.9)	0.320
Cerebral ischemia^a^	10 (7.4)	12 (6.1)	0.402	8 (6.3)	4 (5.7)	0.563

^a^ Values given as *N* (%).

^b^ Values given as median (1st, 3rd quartile); PA—physical activity; mRS—modified Rankin Scale; CeAD—spontaneous cervical artery dissection; NIHSS—National Institutes of Health Stroke Scale.

Those reporting a change in physical activity more frequently had cerebral ischemia as well as occlusive CeAD‐related vessel pathologies at baseline. In addition, those reporting a change in activity habits upon follow‐up had worse mRS and NIHSS scores at admission and longer duration of hospital stays compared to those who did not change activity levels. Older age was the sole characteristic differing in those who decreased compared to those who increased physical activity upon follow‐up, with those increasing activity being younger (Table 1). Of note, 100 of the 197 patients that changed physical activity over time belonged to the Innsbruck cohort, wherein reasoning for change was available. A total of 78 of 100 (78%) of these individuals indicated that CeAD was the prime reason for the change.

Overall, of our 333 included patients, 17 (5.1%) had recurrent CeAD and 22 (6.6%) cerebral ischemia (6 [27.3%] due to recurrent CeAD) within the 6.5 year follow‐up, with outcome events occurring 45.0 months (IQR 29.0, 93.4) and 12.0 (IQR 0.3, 105.7) months, respectively, after index CeAD. There was no difference in the univariate comparison of outcomes between our groups of interest (i.e., patients with and without change or those with an increase or decrease in physical activity, Table 1).

**FIGURE 1:**
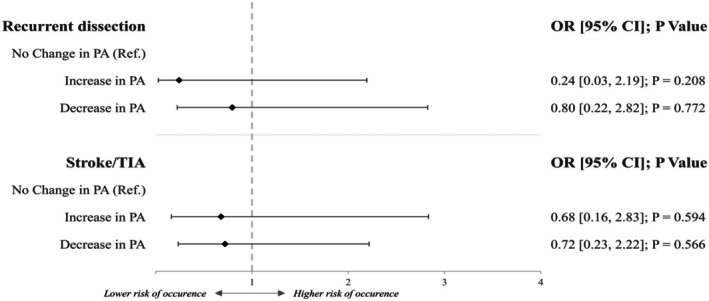
Impact of change in physical activity (PA) on recurrent dissection or stroke upon follow‐up.

Figure 1 depicts the impact of physical activity change on our two outcome parameters in logistic regression analysis. After adjusting for confounders (ischemia at baseline, sex, mRS at admission, CeAD‐related vessel pathology, length of follow‐up, and age), no association between increase or decrease of physical activity to either CeAD recurrence or occurrence of cerebral ischemia upon follow‐up was seen. Concerning ischemic events, results remained unchanged if only those occurring ≥ 1 month (*n* = 14; increase OR 0.79 [0.15, 3.25]; decrease OR 0.81 [0.21, 3.05]) as well as ≥ 3 months (*n* = 13; increase OR 0.78 [0.16, 3.03]; decrease OR 0.86 [0.27, 3.12]) after initial CeAD admission were used.

Baecke score was available in 115 of our 333 (34.5%) included patients, in which 5 (4.3%) recurrent CeAD occurred. Figure S1 presents an overview of physical activities and their respective intensity ratings, as well as a practical guide on the Baecke score sports index calculation. Table 2 presents additional data on sports and levels of exertion within this subgroup of patients with available Baecke score values.

**TABLE 2:** Data on physical activity performed in subgroup of patients with available Baecke score.

*N*	115
Highest intensity of sport performed during follow‐up	
Low intensity	15 (13.0)
Medium intensity	68 (59.1)
High intensity	32 (27.8)
Sports index (*n* = 100)	2.4 (1.9, 3.0)

^a^ Values given as *N* (%).

^b^ Values given as median (1st, 3rd quartile).

**FIGURE 2:**
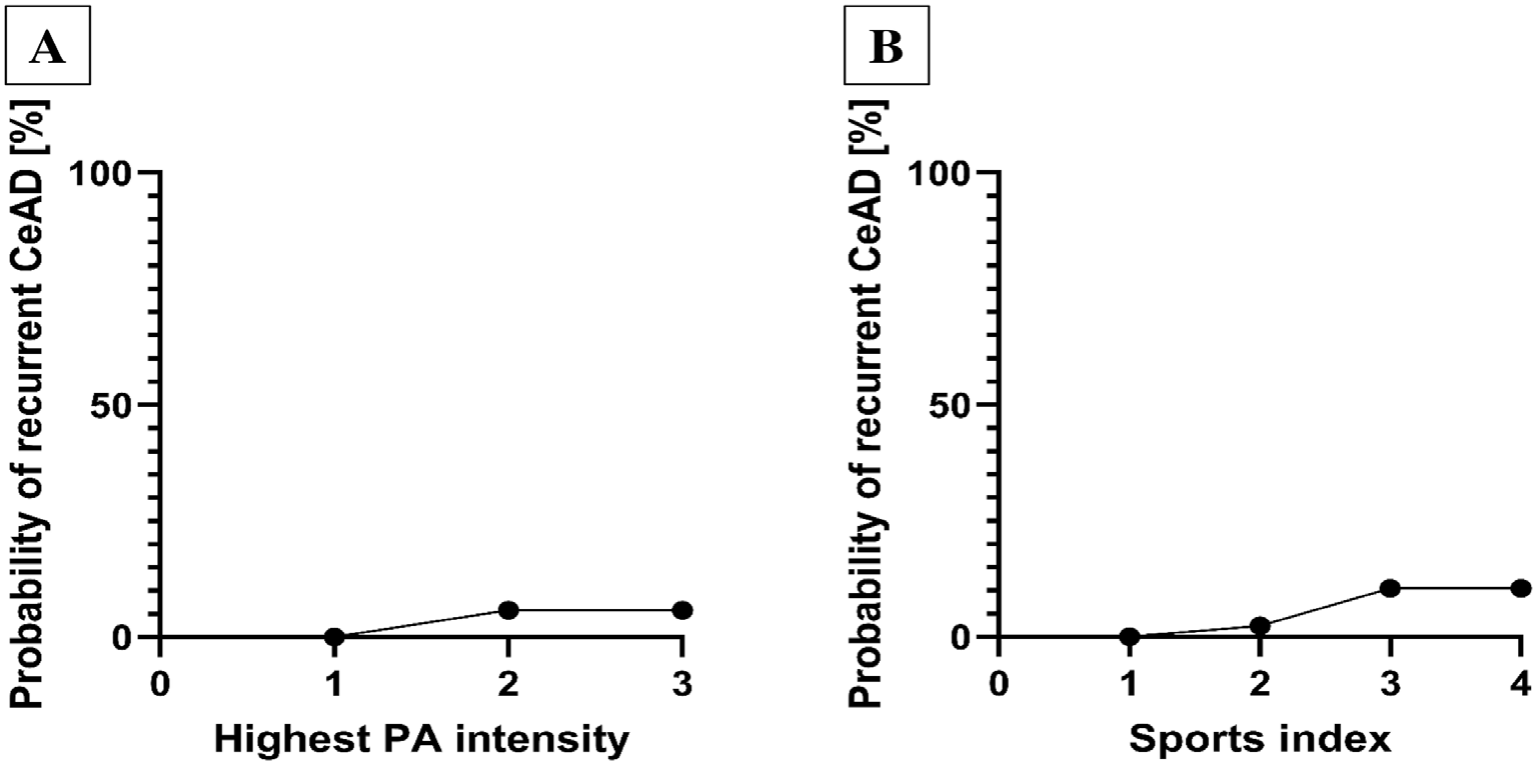
Dose–response relationship between CeAD recurrence and (A) intensity of physical activities (PA) according to type of sport as well as (B) Baecke score ‐derived sports index.

Using these data, sole performance of higher‐intensity physical activity did not have an association with recurrent CeAD (OR 0.49 [95% CI 0.11, 2.32]; *p* = 0.372, Figure 2A). This was also true when using Baecke score‐derived sports index, therefore including regularity of physical activity performed, where no significant association but a trend toward higher sports index values being associated with CeAD recurrence was seen (OR 3.43 [95% CI 0.86, 13.6]; *p* = 0.080—Figure 2B).

## 4 Discussion

Key findings of our study are that (i) in our cohort of 333 CeAD patients with detailed information on physical activity pattern post‐CeAD, more than half (59.2%) report changes in physical activity a median of 6 years after CeAD. More than ¾ of these individuals indicated that CeAD was the prime cause for said change (78 of 100 in subgroup with available data). (ii) A change in physical activity post‐CeAD did not relate to increased risk of dissection recurrence or occurrence of cerebral ischemia upon follow‐up, independent of whether activity increased or decreased (Figure 1). (iii) Still, there was low statistical evidence for a trend toward CeAD recurrence in those regularly performing high‐intensity physical activities (Figure 2).

Prior to our study, it was unclear how to counsel patients concerning physical activity after suffering from CeAD [11]. As minor trauma prior to CeAD is common and frequently occurs during sporting activities, both medical doctors and patients were lacking guidance on which activities or intensities of physical exertion can be considered safe and from which to refrain [4, 8]. Our analysis of a large, representative, unselected cohort of patients treated at centers with considerable expertise in CeAD care emphasizes this conundrum as more than half of individuals changed their physical activity long after CeAD (Table 1). A large number of individuals cited CeAD as the prime reason for this change. Sufficient physical activity over time is known to be associated with reduced risk of stroke [21]. In all types of stroke survivors, physical deconditioning and sedentary lifestyles are highly prevalent and relate to worse outcomes as well as hampered social reintegration [22, 23]. This has led to calls for inclusion of physical activity recommendations into post‐stroke care [22]. Therefore, the large number of patients reporting a decrease in physical activity after suffering CeAD, one of the main reasons for stroke in the young, is concerning and emphasizes the need for evidence on the safety of physical exertion after CeAD. Our study indicates that neither decrease, increase nor sole performance of high‐intensity physical activity are associated with a higher risk of recurrent CeAD or occurrence of cerebral ischemia in the long run (Figures 1, 2A). However, if high‐intensity sports are performed at a higher rate, which relates to higher values in the Baecke score‐derived sports index, a possible trend toward increased risk of recurrent CeAD can be reported (OR 3.43 [0.86, 13.64]; *p* = 0.080—Figure 2B).

Consequently, our data suggest that patients should not be discouraged from physical activity. However, it seems that the frequency of high‐intensity physical activity should be discussed and, adhering to the limited data available, adherence to moderate‐intensity levels should be recommended. We acknowledge that our study has limitations. One being that the change in physical activity depends on patient‐reported activity levels without more objective measures of activity prior to CeAD. Further, the patients were tasked to recall their physical activity level and sports performed during the median follow‐up of 6 years, delivering an estimate over time. The issue of reporting as well as recollection bias and the possibility that participants provided false or exaggerated accounts of their physical activity were not specifically addressed. Further, reason for change in physical activity over time was not consistently recorded within our study cohort. It would be fruitful to address changes in needs, fears, or physical barriers in more detail, preferably within a prospective study, to paint the entire picture. A key aspect in this sense would also be to explicitly assess physical activity prior to CeAD recurrence, which was not available as an additional variable in our dataset. Additionally, specific sporting activities are presumed to be associated with a higher risk of CeAD, such as contact sports (e.g., hockey and combat sports) or those entailing rapid head movements or prolonged head extension. As contact/combat sports were rare within our cohort, we cannot draw reliable conclusions, making future assessments, which additionally encapsulate a more mechanistic approach, rewarding. A regional bias on types of sports done can also be presumed as our study encompasses two patient cohorts from regions known for winter sports and hiking. This is also true for Baecke score and reasons for change in physical activity reported as these data are limited to the Innsbruck (ProSect) cohort. Lastly, we emphasize the fact that our data have to be interpreted with caution as it is purely observational and, due to the overall low number of CeAD recurrences in our cohort, reliability of results may be limited. However, our results call for future analyses with a larger sample size, especially concerning physical activity intensity.

In all, through the analysis of our large CeAD cohort study, we can emphasize that CeAD patients should be reassured that regaining physical activity after CeAD can be considered safe. However, more in‐depth data on safety limits concerning levels of physical exertion are needed, which calls for future studies with a larger sample size.

## Author Contributions

**Benjamin Dejakum:** investigation, writing – review and editing, resources. **Josefin Kaufmann:** conceptualization, investigation, writing – original draft, methodology, visualization, writing – review and editing, formal analysis, project administration, data curation.

## Funding

Lukas Mayer‐Suess and Michael Knoflach's research was funded by the Österreichische Nationalbank Anniversary fund (#15644). This study is supported by VASCage—Research Centre on Clinical Stroke Research. VASCage is a COMET Centre within the Competence Centers for Excellent Technologies (COMET) programme and funded by the Federal Ministry for Climate Action, Environment, Energy, Mobility, Innovation and Technology, the Federal Ministry of Labour and Economy, and the federal states of Tyrol, Salzburg and Vienna. COMET is managed by the Austrian Research Promotion Agency (Österreichische Forschungsförderungsgesellschaft). FFG Project number: 898252. This work was supported by Oesterreichische Nationalbank, 15644 Österreichische Forschungsförderungsgesellschaft, 898252.

## Ethics Statement

All analyses were approved by the local ethics committee at the Medical University Innsbruck (EK#UN5072,325/4.1) and of Basel (EKNZ PB‐2016‐00547). Appropriate informed consent of patients who took part in both registries was obtained.

## Conflicts of Interest

The authors declare no conflicts of interest.

## Supporting information


**Figure S1:** Baecke Score‐derived sports index (PA—physical activity).

**Table S1:** Differences between those with and without physical activity (PA) data available.

## Data Availability

The data that support the findings of this study are available from the corresponding author upon reasonable request.
